# Vagus nerve stimulation in *KCNB1*-related developmental and epileptic encephalopathy: A case of seizure reduction and review of literature

**DOI:** 10.1016/j.ebr.2025.100826

**Published:** 2025-08-30

**Authors:** Taichi Sayanagi, Kenzo Kosugi, Eri Ogawa, Toshiki Takenouchi, Mamiko Yamada, Kenjiro Kosaki, Ichiro Sugiyama, Masahiro Toda

**Affiliations:** a Department of Neurosurgery, Keio University School of Medicine, 35 Shinanomachi, Shinjuku-ku, Tokyo 160-8582, Japan; b Department of Pediatrics, Keio University School of Medicine, Tokyo, Japan; c Department of Child Neurology, Okayama University Graduate School of Medicine, Dentistry and Pharmaceutical Sciences and Okayama University Hospital, Okayama, Japan; d Center for Medical Genetics, Keio University School of Medicine, Tokyo, Japan; e Department Head, Department of Neurosurgery, Kyosai Tachikawa Hospital, Tokyo, Japan

**Keywords:** KCNB1-related DEE, DEE, VNS, Refractory epilepsy, Fenfluramine, EEG

## Abstract

Developmental and epileptic encephalopathies (DEEs) are devastating syndromes characterized by early-onset, treatment-resistant seizures, and significant developmental delays. *KCNB1*-related DEE, arising from pathogenic variants in the *KCNB1* gene, presents with profound cognitive impairment and persistent seizures despite multiple antiseizure medications (ASMs). Vagus nerve stimulation (VNS) has emerged as a valuable adjunctive therapy for medically intractable epilepsy, yet its effectiveness in rare genetic DEEs remains unknown. We report the first case of VNS in a 5-year-old child with *KCNB1*-related DEE, achieving about 90% seizure reduction after one year. Prior treatments with multiple ASMs had minimal effect. After VNS implantation, optimized stimulation parameters combined with fenfluramine markedly improved seizure control. Although concurrent medication adjustments and transient seizure cessation during infection complicated interpretation, the sustained seizure improvement strongly suggests VNS’s efficacy in this genetic epilepsy.

This case illustrates VNS’s potential benefit and possible synergy with fenfluramine in *KCNB1*-related DEE. Further accumulation of case reports is necessary to confirm these findings.

## Highlights

• First report of VNS for KCNB1‐related DEE in a pediatric patient.
• Achieved 90% seizure reduction at one-year follow-up.
• Post‐VNS EEG showed resolution of epileptiform discharges at 6 months.
• Optimized VNS parameters with fenfluramine indicate a synergistic effect.
• Supports multimodal treatment for rare, refractory genetic epilepsy.

## 1 Introduction

Developmental and epileptic encephalopathies (DEEs) are severe neurological disorders presenting with early-onset, refractory seizures, and profound developmental impairment [1]. Among these conditions, *KCNB1*-related DEE is caused by variants in the *KCNB1* gene, which encodes the Kv2.1 voltage-gated potassium channel [2,3]. Patients often exhibit treatment-resistant epilepsy and severe cognitive deficits, and identifying effective therapies remains challenging [3,4].

Vagus nerve stimulation (VNS), a palliative therapy involving intermittent electrical stimulation of the vagus nerve, has shown promise in various forms of refractory epilepsy [5,6]. Recent studies have explored VNS parameter settings and mechanisms in neurological and psychiatric disorders [7,8], and VNS has demonstrated efficacy in some refractory epilepsies, reducing seizure frequency and severity [5,6]. However, its utility in rare genetic epilepsies such as *KCNB1*-related DEE remains insufficiently explored.

Here, we present the first reported case of VNS implantation in a patient with *KCNB1*-related DEE, significantly reducing seizure frequency at one-year follow-up. This report broadens the therapeutic landscape and highlights VNS as a potential component of a complex, multimodal treatment approach.

## 2 Case report

**Fig. 1:**
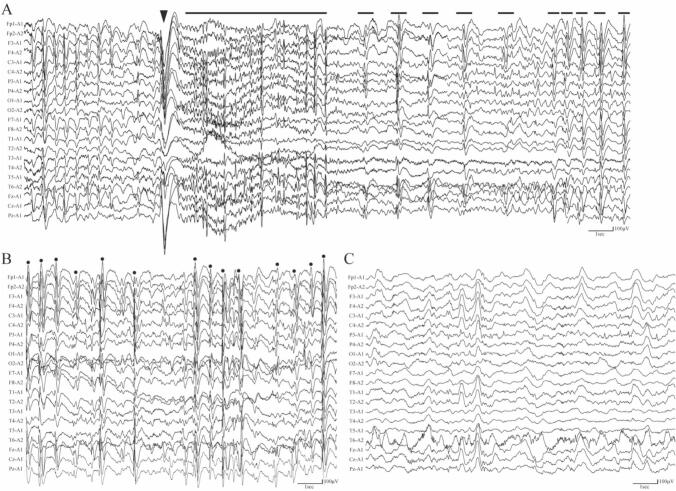
Representative electroencephalographic tracings obtained with referential montages are shown for pre- and post-VNS conditions. Preoperative, ictal EEG was obtained while the patient was awake, preoperative and postoperative interictal EEG was obtained while the patient was asleep. (A) Pre-VNS ictal EEG: The black triangle (▲) marks seizure onset with a myoclonic jerk, followed by a tonic phase (solid black bar) and a clonic phase (dashed line). (B) Pre-VNS interictal EEG: Black circles (●) indicate frequent generalized spike-wave discharges. (C) Six months post-VNS interictal EEG: Note the absence of epileptiform discharges compared to the pre-VNS baseline.

A 5-year-old patient with refractory epilepsy and global developmental delay was referred for surgical evaluation. Seizures began at one year of age, and carbamazepine (CBZ) was initiated thereafter, but the tonic-clonic seizure (TCS) frequency continued to increase. CBZ was switched to valproic acid (VPA) at 2 years, and this relieved the frequency of the TCS by a little but eventual got worse. Clobazam (CLB) was added at 2.5 years; zonisamide (ZNS) introduced at 3 years of age, both medication showing mild decrease in the frequency of TCS. At 3.5 years, CLB was replaced by levetiracetam (LEV) due to sleepiness, and genetic testing identified a *de novo* heterozygous *KCNB1* missense variant (NM_004975.4:c.949C > T; p.(Leu317Phe)). Based on this finding, the patient was diagnosed with DEE associated with a *KCNB1* mutation. ZNS was tapered and lacosamide (LCM) was added at 4.5 years. By age 5, the patient remained on VPA (20 mg/kg/day), LEV (50 mg/kg/day), and LCM (8 mg/kg/day) with persistent seizures and showed developmental regression. At this point, the patient was having four to five myoclonic-tonic-clonic seizures and 30 myoclonic seizures per day. MRI revealed diffuse cerebral atrophy without focal lesions. EEG demonstrated generalized spike-and-wave discharges interictally and generalized myoclonic-tonic-clonic seizures ictally (Fig. 1**A, B**). With no resectable lesion, VNS was chosen as a palliative intervention and the stimulator was implanted without complications.

Postoperatively, VNS output current was gradually increased, and the LCM dose was adjusted. Four months after implantation, the patient developed influenza, resulting in a transient, complete cessation of seizures. Seizures ceased completely for approximately three months after the influenza infection; this seizure-free period was based on the account of the patient's caregivers, who were with the child throughout the day. During this period, however, marked hyper-irritability with inconsolable crying, reduced sleep, and motor restlessness emerged. Considering possible LEV-related symptoms, LEV was tapered off, leading to an improvement in behavioral symptoms but a recurrence of seizures; myoclonic-tonic-clonic seizure frequency at recurrence was approximately one or two times per week, and myoclonic seizure was observed four-five times per day, which is improved compared to the preoperative baseline by more than 80 % for both seizure types. A six-month post-VNS EEG showed no epileptiform discharges (Fig. 1**C**).

**Fig. 2:**
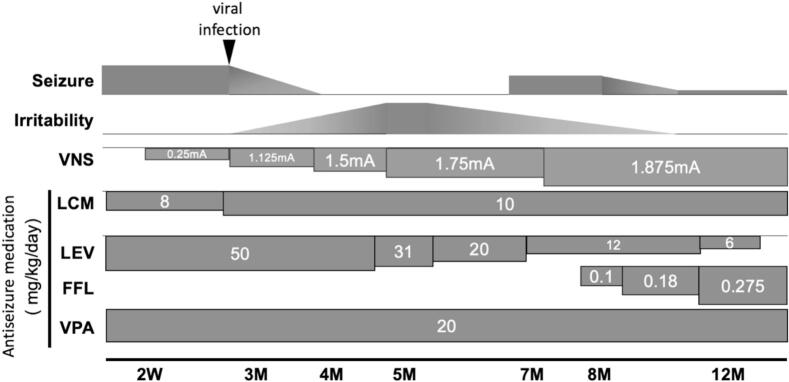
Postoperative clinical course following VNS implantation. The horizontal axis represents time after surgery. The top section displays seizure frequency and irritability over the observation period. A transient cessation of seizures occurred following a viral infection (arrow), coinciding with changes in irritability levels. Below, the black line indicates the incremental adjustments to VNS output current (from 0.25 mA to 1.875 mA). The subsequent bands show the timeline of antiseizure medication (ASM) dosages, including lacosamide (LCM), levetiracetam (LEV), fenfluramine (FFL), and valproic acid (VPA). Dosage changes and tapering are indicated by variations in bar length and shade, with numerical values representing daily doses. By 12 months postoperatively, a ∼90 % reduction in seizure frequency was achieved compared to the preoperative baseline.

Recognizing features such as slow spike-wave interictal discharges and generalized paroxysmal fast activity on ictal EEG (Fig. 1**AB**), plus daily tonic seizures which overlaps with Lennox-Gastaut syndrome, fenfluramine (FFL) was introduced, and further VNS parameter optimization was performed. At one year, seizure frequency had decreased by more than 90 % compared to baseline for both seizure types. (myoclonic-tonic-clonic seizure frequency was approximately once a month, and myoclonic seizure was observed once every two days). Despite the global developmental delay, there was no regression after VNS implantation. Shortly after initiating VNS therapy, the patient showed improvements in emotional and affective aspects, including an increased frequency of smiling and vocalization, as observed by caregivers. Also, motor development improved, and he could roll over and sit without support by the age of 6 years and 6 months. The timeline of seizure frequency, irritability, VNS adjustments, and ASM changes is summarized in Fig. 2.

## 3 Discussion

*KCNB1*-related DEE poses a significant therapeutic challenge due to its early onset, profound developmental impact, and marked pharmacoresistance [[2], [3], [4]]. This case highlights the complexity of attributing clinical improvement to a single intervention, as multiple treatments—including LCM escalation, LEV tapering, and FFL introduction—occurred alongside VNS. However, the observed reduction in both seizure frequency and severity before FFL initiation may support an independent beneficial effect of VNS therapy in this patient since LCM escalation did not seem to have strong impact on the seizure frequency.

Although we acknowledge that concurrent initiation of FFL likely contributed substantially to the significant seizure reduction ultimately observed, the evident improvement in seizure frequency prior to its introduction suggests that VNS therapy itself played a meaningful role. Indeed, neuromodulation by VNS may have provided a more favorable neurological milieu, enhancing responsiveness to subsequently introduced treatments such as fenfluramine.

VNS exerts its effects by modulating neuronal excitability and altering cortical network activity [7,8]. Previous reports have demonstrated meaningful seizure reductions in various refractory epilepsies, though complete freedom from seizures is less common [5,6]. In rare genetic DEEs, the literature is scarce, making this first report in a *KCNB1*-related DEE patient particularly noteworthy.

While behavioral changes such as irritability and excitability were observed, these might reflect improved arousal and cognition or complex interactions between ASM regimens and altered cortical excitability. Notably, transient psychiatric manifestations, including mania following VNS, have been reported [9]. Although mania was not observed in our patient, the literature underscores the need for close monitoring of mood and behavior in patients undergoing VNS therapy.

The transient seizure cessation following influenza infection adds another layer of complexity. Several reports describe spontaneous improvements in intractable childhood epilepsy following acute viral infections [10]. Similarly, transient improvements in conditions like Infantile Spasms Syndrome have been documented during febrile episodes [11]. Although the precise mechanisms remain unknown—potentially involving immunological, metabolic, or inflammatory factors—these episodic changes emphasize the multifactorial nature of seizure control and caution against attributing improvement solely to any single intervention, including VNS.

Despite these complexities, the long-term outcome—approximately 90 % seizure reduction in one year—exceeds the preoperative baseline and suggests a substantial positive impact. Importantly, VNS did not worsen the patient’s condition and may have created a more favorable neurological milieu that allowed other treatments, like FFL, to have a greater effect.

The landscape of VNS efficacy in monogenic DEEs is varied, with more extensive literature and *meta*-analyses available for certain conditions compared to others where evidence may still be emerging, such as for some rare gene variants. For instance, the analyses have provided consolidated insights into VNS outcomes for conditions like Dravet syndrome and Rett syndrome, offering valuable benchmarks.

Although the literature on VNS usage for *KCNB1*-related DEE is scarce, there are retrospective data from larger cohorts providing compelling evidence supporting the efficacy of VNS in managing DEEs. Two key studies form the cornerstone of this analysis: one involving 32 children with DEE [12] and a Norwegian population-based study encompassing a broader cohort of DEE patients [13]. Together, these studies offer important insights into the magnitude of seizure reduction, predictors of response, and the differential impact of VNS on various seizure types. In the study by Geng et al., responder rate, defined as achieving a ≥ 50 % reduction in seizures, was observed in 68.8 % of the patients at the most recent follow-up. The Norwegian study led by Kostov et al. reported that 37.1 % of patients with DEE achieved a ≥ 50 % reduction in seizures 24 months after VNS implantation. This improvement is particularly remarkable given the inherently refractory nature of DEEs, which are often resistant to multiple ASMs. The present case demonstrated several favorable prognostic indicators for a positive response to VNS therapy, including normal MRI findings and the absence of epileptic spasms [12]. Other *meta*-analyses have indicated that the overall responder rate reached 55 % for patients with Dravet syndrome, particularly those with a shorter duration of epilepsy (less than six years) [14]. Similarly, a responder rate of 54 % was observed for patients with Lennox-Gastaut syndrome [15]. A small cohort demonstrated the efficacy of VNS in cases of rare drug-resistant epilepsy with monogenic etiology, showing a responder rate of 55 % [16]. It is noteworthy that comments on combined use of fenfluramine or the proportion of patients who exhibited more than 90 % reduction in seizures, as seen in our case, were not described in the literature.

While further studies and the accumulation of more case data are needed to clarify the precise role of VNS in *KCNB1*-related DEE and other rare genetic epilepsies, this case report provides valuable insights in elucidating the mechanism of action of the VNS. The significant improvement in seizure control observed with the combined use of VNS and FFL in this case, compared to previous treatments with conventional ASMs, suggests that the modulation of serotonergic pathways by both interventions [17,18] may be a contributing factor. The fundamental pathology of this disease is thought to involve an elevated intracellular potassium concentration due to dysfunction of potassium channels, a review of current literature did not reveal a direct link between VNS and potassium channel modulation. However, while direct evidence linking VNS to the modulation of specific potassium channels such as Kv2.1 remains limited, it has been reported that VNS can increase the expression of calcium-activated potassium channel [19] in the nervous system, consequently promoting potassium efflux and neuronal hyperpolarization, thus contributing to an inhibitory effect.

This is the first report of a patient with *KCNB1*-related DEE undergoing VNS therapy, resulting in a significant and sustained reduction in seizure frequency after one year. While concurrent treatment changes prevent definitive attribution of improved seizure control solely to VNS, our findings support considering VNS as part of a multimodal approach. Further accumulation of case reports on VNS therapy for *KCNB1*-related DEE is warranted.

## CRediT authorship contribution statement

**Taichi Sayanagi:** Writing – review & editing, Writing – original draft, Formal analysis, Data curation, Conceptualization. **Kenzo Kosugi:** Writing – review & editing, Writing – original draft, Formal analysis, Data curation, Conceptualization. **Eri Ogawa:** Data curation. **Toshiki Takenouchi:** Data curation. **Mamiko Yamada:** Investigation, Formal analysis. **Kenjiro Kosaki:** Investigation, Formal analysis. **Ichiro Sugiyama:** Validation. **Masahiro Toda:** Validation, Supervision.

## Funding

This report was not supported by any sponsor or funder.

## Declaration of competing interest

The authors declare that they have no known competing financial interests or personal relationships that could have appeared to influence the work reported in this paper.
